# Minds, Brains, and Capacities: Situated Cognition and Neo-Aristotelianism

**DOI:** 10.3389/fpsyg.2020.566385

**Published:** 2020-12-21

**Authors:** Hans-Johann Glock

**Affiliations:** Department of Philosophy, Center for the Interdisciplinary Study of Language Evolution (ISLE), University of Zurich, Zurich, Switzerland

**Keywords:** situated cognition, neo-aristotelianism, brain, mereological fallacy, capacities, criteria, Wittgenstein

## Abstract

This article compares situated cognition to contemporary Neo-Aristotelian approaches to the mind. The article distinguishes two components in this paradigm: an Aristotelian essentialism which is alien to situated cognition and a Wittgensteinian “capacity approach” to the mind which is not just congenial to it but provides important conceptual and argumentative resources in defending social cognition against orthodox cognitive (neuro-)science. It focuses on a central tenet of that orthodoxy. According to what I call “encephalocentrism,” cognition is primarily or even exclusively a computational process occurring inside the brain. Neo-Aristotelians accuse this claim of committing a “homuncular” (Kenny) or “mereological fallacy” (Bennett and Hacker). The article explains why the label “fallacy” is misleading, reconstructs the argument to the effect that encephalocentric applications of psychological predicates to the brain and its parts amount to a category mistake, and defends this argument against objections by Dennett, Searle, and Figdor. At the same time it criticizes the Neo-Aristotelian denial that the brain is the organ of cognition. It ends by suggesting ways in which the capacity approach and situated cognition might be combined to provide a realistic and ecologically sound picture of cognition as a suite of powers that flesh-and-blood animals exercise within their physical and social environments.

## Introduction

Situated cognition constitutes a powerful trend in contemporary cognitive science. One of its pillars is a fresh approach to philosophical problems concerning the very nature of the mental. More specifically, situated cognition raises questions about the ontology of cognition. What are the subjects of cognitive properties, states, and processes? What is the proper locus of cognition? Is cognition confined to the brain, or is it situated in whole bodies, organisms, and perhaps their environments?

Orthodox cognitive science is representationalist and computationalist. It treats cognition as a matter of calculations performed on symbolic or sub-symbolic representations. Both the representations and the computations operating on them are supposed to be implemented *inside the brain*. Accordingly, this orthodoxy subscribes to what I call “encephalocentrism”^1^. By contrast, situated cognition is part of an anti-subjectivist paradigm shift which also operates under the label “4E Cognition” (Newen et al., 2018b). Cognition is *embodied* in that it is not confined to the brain, but involves the whole subject. It is *embedded* in that it is essential to cognition that this embodied subject is situated in a physical and social environment. It is *enactive*, in that it is equally essential that the subject operates actively within its environment, even in allegedly passive processes like perception. It is *extended* in that cognition may reach beyond the limits of the body, to features of the environment which are employed in understanding and explanation.

The impetus for this article is provided by the fact that there are venerable ancestors to situated-cum-4E cognition. More importantly, these ancestors have spawned contemporary work that is in many respects congenial to situated cognition, yet these parallels have so far gone largely unnoted^2^. Most importantly, attending to both the convergences and the differences sheds important light on the nature of cognition, and it holds the promise of overcoming encephalocentric opposition to situated cognition.

The ancestors and cousins of situated cognition that I have in mind are Aristotelian and/or Wittgensteinian currents within the philosophy of mind. To simplify matters, I shall henceforth speak of “Neo-Aristotelianism.” Whereas, Wittgenstein himself revived the Aristotelian-cum-Thomist tradition unwittingly, others (Ryle, Anscombe, Geach) did so knowingly. Having been sidelined by the representationalist and computationalist mainstream since the 1960s, their perspective has been rehabilitated through the rediscovery of dispositions and abilities (Kenny, 1989; Hacker, 2007; Vetter, 2015; Schellenberg, 2018).

Neo-Aristotelianism revolves around a “capacity approach”: a mind is neither a physical nor a mental substance, but a set of abilities which can be attributed and understood from a third-person perspective. This general parallel to the anti-subjectivist stance of situated cognition is supplemented by more specific parallels concerning the ontology of cognition. According to Neo-Aristotelianism it is neither a non-material soul nor a part of the body that cognizes—feels, desires, perceives, conceptualizes, thinks, infers, etc. Instead, it a *whole flesh-and-blood animal*—human or non-human—operating in its physical and social surroundings.

In this context, Neo-Aristotelianism challenges major tenets of the encephalocentrist orthodoxy. Encephalocentrism can take various forms. We must distinguish the claim that the brain and its parts are *subjects* of cognition—the things which possess cognitive properties, are in cognitive states or undergo cognitive processes—from the claim that they are the *location* of cognition. Since neither properties nor states have a spatial location, that second claim should be restricted to cognitive processes and activities. Next, one might distinguish *homuncular* from *non-homuncular* encephalocentrism. According to “homuncular functionalism,” there are human-like agents in the brain that perform acts of cognizing such as computation and inference [see Lycan (1991)] and section Behavior and the brain below). But even according to non-homuncular functionalism, the property of undergoing a mental process/being in a mental state is the property of undergoing a neurophysiological process/being in a neurophysiological state. Since the subject of such neurophysiological processes and states is the brain or one of its parts, this implies that the latter is also the subject of mental processes and states. Finally, encephalocentrism can be more or less pronounced. A *strong* version has it that only the brain and its parts are subjects of cognitive states, processes, and activities; and all cognition takes place within the skull. Thus, according to classic functionalism, the cognitive system interacts with the environment, yet cognition is nonetheless completely realized by computational processes in the brain. A *weaker* version maintains only that the brain and its parts are among the subjects of cognition, and that some cognitive processes take place within the brain. Neo-Aristotelians take issue with all of these positions. Any ascription of cognitive states or processes to the brain is found guilty of a “homunculus fallacy” (Kenny, 1984, ch. 9) or a “mereological fallacy” (Bennett and Hacker, 2003; Maslin, 2006). In response, Dennett (2007) and Searle (2007) have defended weak encephalocentrism. Recently, there has also been a more radical reaction, according to which even demanding cognitive concepts apply literally to just about any biological phenomenon, basic physiological components of organisms included (Figdor, 2018).

If encephalocentrism could be vindicated in the face of Neo-Aristotelian criticism, the path to situated cognition would be blocked. For even weak encephalocentrism explains the cognitive powers and performances of whole animals by reference to *cognitive* powers and performances of their neurophysiological components. Accordingly, the *real subjects* of the fundamental cognitive states and processes are parts of individual organisms, and the *ultimate location* of cognition is within our skulls^3^. This would imply that situated cognition is at most a *derived* phenomenon. While it is the animal as a whole which is situated and operates within a material and/or social context, its cognitive exploits and abilities would be fully explicable by reference to its parts (organs). Even if the rest of the body and the activities of the whole animal within its environment must be taken into account, they have a bearing only through their impact on the cognitive phenomena in the brain. Their role can be accommodated within the encephalocentric orthodoxy^4^.

My contribution propounds a critique of encephalocentrism that sets store by the capacity approach while relinquishing other aspects of Neo-Aristotelianism. It starts out by indicating why an open-minded Wittgensteinian approach is preferable to Aristotelian essentialism, especially when it comes to linking up with situated cognition. Next, it argues that the labels “homuncular fallacy” and “mereological fallacy” are inaccurate, since the fundamental bone of contention is whether attributing cognitive and epistemic processes and abilities to organs and their parts amounts to a category mistake. On that issue, I side with the “Nonsense View” of Bennett and Hacker. I reconstruct the argument behind it and defend modified versions of its premises against animadversions by Searle and Dennett. Next I rebut Figdor's frontal attack on the Nonsense View. She appeals to current versions of encephalocentrism, such as predictive processing. I criticize her philosophical case for holding that these positions vindicate a literal interpretation of attributing cognition to the brain; yet it is not my ambition to demonstrate that they could only make a coherent contribution to neuroscience if they could be taken literally^5^. Instead, the next section concludes the philosophical dialectic: denying that the brain is the organ of cognition takes opposition to encephalocentrism too far. I end by summing up how the capacity view and situated cognition can benefit from each other.

## The Neo-Aristotelian Framework

Neo-Aristotelians engage in a priori reflections of a metaphysical or conceptual kind. Situated cognition, by contrast, presents itself as thoroughly empirical and hostile to “armchair philosophy.” It addresses the same questions concerning the nature and locus of cognition, “what” cognition is and “where” it takes place. But among its champions “there is a general agreement that a priori definitions and models of cognition are not helpful, and that we need to conduct experiments and consult the empirical literature” (Newen et al., 2018a, 9).

Fortunately, these two options are neither exhaustive nor incompatible. Empirical investigations no less than philosophical reflections rely on at least a preliminary understanding of what topic is being investigated. And we identify these topics through our established concepts, whether everyday, scientific or philosophical. These concepts are presupposed explicitly or implicitly not just by philosophical theories and arguments, but also by research projects, methods, and findings within the special sciences (see section Technical Uses and Metaphor).

Psychological notions are notorious for giving rise to a whole raft of vexatious puzzles. Prominent among them are the mind-body problem, the “riddle of consciousness,” and the mark and scope of the cognitive, which is our topic. Any sober approach even to scientific problems concerning the mind should therefore pay attention to the established employment of the relevant expressions within their normal surroundings. Without the propaedeutic of conceptual clarification, we shall be “incapable of discussing the matter in any useful way because we have no stable handle on our subject matter” (Joyce, 2006, 52). Furthermore, we shall be liable to fallacies and confusions because of illicitly oscillating between different senses of pertinent expressions.

At the same time, such conceptual clarifications must be sensitive to the way in which concepts are understood and operationalized in scientific research programs and to their modification in the face of novel observational and experimental data (see Glock, 2017 and section Conclusion below). By contrast to Aristotelian essentialism, the Wittgensteinian strand in Neo-Aristotelianism acknowledges that our concepts are untidy and subject to change. This attitude is hospitable to a key ambition of situated cognition, namely to construct novel conceptual and methodological frameworks for the empirical study of cognition. Wittgensteinianism is also at odds with a Neo-Aristotelian tendency to equate the mind with the intellect (Kenny, 1989, 20–5; Hacker, 2013, ch. 1). Instead, it treats “mind,” “mental,” and their cognates as family-resemblance concepts. The phenomena they signify are united not by a single common feature, but by a complex network of overlapping and criss-crossing similarities (Wittgenstein, 1953, §§66–7). Such an approach supports another prominent conviction among proponents of situated cognition: there is no single “mark of the mental” (Newen et al., 2018a, 7, 10).

## What Capacities Can Do for Understanding Cognition

Even if Neo-Aristotelianism goes wrong in seeking to identify an immutable essence of the mind, its *capacity approach* is both enlightening and congenial to situated cognition. In the wake of Descartes, the mainstream of Western philosophy has treated “mind,” its equivalents and cognates as the label of a special kind of thing, whether it be a separate mental substance, as in dualism, or the brain, as in materialism. The starting point of the capacity approach is negative: the mind is not a *bona fide* thing of any kind, whether mental–a *res cogitans*—or material—the brain (see White (1972), 464–5). Nor is it a kind of stuff or matter like water or gold: “mind” is a count-noun, and hence unsuitable as the name of a stuff.

The capacity approach also offers an alternative, by regarding the mind as a potentiality or power (Hacker, 2007, 90–121; Kenny, 2010). Potential properties are *bona fid*e attributes of particulars and substances, contrary to various forms of reductionism. At the same time, a potentiality must not be reified, treated as a thing of a peculiar kind that somehow co-exists with the particular or substance that possesses it. A power is neither a fiction, nor a flimsy actuality, nor an ethereal substance.

The central lesson: whether a subject has mental properties depends on what she is capable of doing. And to have a mind is to have a range of cognitive, volitional, and affective capacities or abilities. These must not be confused with

1. their exercise (in bringing about or undergoing physical or mental change);
2. the conditions that must obtain for manifesting or exercising the ability:
  - opportunity conditions: I may be able to dissect an angle with compass and ruler, yet lack the necessary equipment.
  - enabling conditions: I may possess that ability and have the prerequisites but be impeded by disease (e.g., high fever) or injury (e.g., fractured hands).
3. their possessor (the individual animal or person);
4. their vehicle, that is the physical ingredient or structure of the possessor that sustains the ability, i.e., causally enables the possessor to exercise it (subject to opportunity and enabling conditions).

Judged from this perspective, dualism reifies mental powers, behaviorism reduces these powers to their exercise, and the mind-brain identity theory reduces them to their vehicle–the brain. Neo-Aristotelians have also relied on the capacity approach to resist encephalocentrism more generally.

## Fallacies and Category Mistakes

There is a venerable tradition of attributing mental and epistemic properties to things other than flesh-and-blood animals. Dualists like Plato and Descartes identify the mind with a non-material substance. For them, it is the soul that thinks (etc.) and thinking occurs “in” the soul. Against Cartesian dualism Wittgenstein insisted:

Only of a human being and what resembles (behaves like) a human being can one say: it has sensations; it sees; is blind; hears; is deaf; is conscious or unconscious (1953, §281).

Alluding to this quote, Kenny criticized the “reckless application of human-being predicates to insufficiently human-like objects,” notably the brain and its parts, in psychology and psycho-linguistics. He labeled this mistake the “homunculus fallacy” (Kenny, 1984, 125). For crediting the brain with perceiving, remembering, understanding, inferring etc. invites the question of how a conglomeration of neurons could do so. Since no convincing answer is forthcoming, one is driven to the absurd conclusion that there are homunculi in the brain who are capable of such cognitive feats.

Bennett and Hacker were in turn inspired by Kenny. But they also invoke “mereology,” “the logic of part/whole relations.” The “mereological fallacy” consists in “ascribing to a part of a creature attributes which logically can be ascribed only to the creature as a whole.” It violates the “mereological principle”: “psychological predicates which apply only to human beings or (other animals) as wholes cannot intelligibly be ascribed to their parts, such as the brain” (Bennett and Hacker, 2003, 29, 73). The Neo-Aristotelians concede that this mistake is

not strictly speaking a fallacy, i.e., an invalid argument, since it is not an argument but an illicit predication. However, it leads to invalid inferences and arguments, and so can be loosely called a fallacy (Smit and Hacker, 2014, 1077; see also Kenny, 1984, 135-6; Bennett and Hacker, 2003, 73n13).

These terminological remarks call out for scrutiny. Note first that the difference between the alleged encephalocentric mistake and a fallacy in the “strict” logical sense does not just concern the level of complexity—a single statement (illicit predication) vs. a set of statements (invalid argument). In the case of a fallacy it is logically possible that the premises should all be true *and* the conclusion nonetheless false. But Bennett and Hacker are adamant that statements to the effect that parts of an animal cognize (perceive, experience, think, infer, etc.) are *nonsensical* rather than false (e.g., Bennett and Hacker, 2003, 2, 6, 72; Bennett and Hacker, 2007, 135). Unlike

(1) Mary cognizes

statements like

(2) Mary's brain / a part of Mary's brain cognizes

“make no literal sense.” By these lights, statements like (2) cannot even be the *conclusion* of a fallacy. For they are bereft of linguistic meaning and hence *neither* true *nor* false.

Next, the Neo-Aristotelian defense of the tag “fallacy” invites a quick and dirty response. Many encephalocentrists concede that statements of form (2) are true not strictly speaking, but only in a loose or extended sense. According to Dennett (2007, 87–8), for instance, brains or their parts can only “sort of believe” (etc.). Bennett and Hacker object that in that case, a statement like (2) is only “sort of true,” only “sort of explains” statements like (1), and only sort of makes sense (Bennett and Hacker, 2007, 140). By the same literalist standards, don't they inveigh against a “sort of fallacy”? The response is too quick, however. Bennett and Hacker's underlying complaint is that Dennett fails to explain what it is for parts of an animal to “sort of believe.” By contrast, Neo-Aristotelians explain, albeit briefly, in what “extended sense” the mistake is a fallacy: it *leads* to fallacies.

Now, their opponents could retort that they can offer an analogous explanation:

(2^*^) Mary's brain / a part of Mary's brain *sort of* cognizes means
(2′) Neurophysiological processes in Mary lead to Mary's cognizing

However, the two cases do not run in parallel. In (2′), “lead” signifies a relation of mechanical causation. But when Neo-Aristotelians accuse encephalocentric predications like (2) of “leading” to logical fallacies, they mean an epistemic relation: (2) seems to vindicate an argument that is in fact invalid. Furthermore, in their story, one and the same subject commits both the illicit predication and the ensuing fallacy. By contrast, the *sub-personal* phenomena supposedly recorded in (2) and (2^*^) causally explain phenomena at the *personal* level recorded in (1), cognitive capacities and their exercises on the part of a whole animal.

But *what fallacy* is encouraged by applying psychological predicates to parts of an animal? Kenny's answer: if one explains cognitive phenomena at the personal level by reference to cognitive phenomena at the cerebral level, e.g., (1) by (2) or (2^*^), this invites the further conclusion:

(3) There are homunculi in Mary's brain that cognize.

But while the mistake is patent, the terminology is incongruent. The move from (2) to (3) is precisely *not* fallacious by Neo-Aristotelian standards. If psychological predicates can only be ascribed to human-like subjects, then from (2) something like (3) *follows*. Kenny does not unmask a logical fallacy; he presents a *reductio ad absurdum* of encephalocentrism. (3) is absurd, irrespectively of whether it as an obvious empirical falsehood or conceptually incoherent.

Bennett and Hacker prefer the label “mereological fallacy” precisely because it does not impute a commitment to homunculi in the brain (2003, 73, fn.13). But their mereological take on the matter is also fraught with difficulties. They recognize that some psychological predicates can apply to parts of an animal, notably verbs of sensation, as in:

(4) Mary's hand hurts.

Accordingly, there is *no general principle* precluding the transfer even of psychological predicates from whole animals to their parts. Moreover, Bennett and Hacker also invoke Wittgenstein's afore-quoted dictum against ascribing psychological properties to objects that are *not* parts of animals, such as plants and computers. In conjunction, these two points show that the encephalocentric mistake cannot be a matter of mereology.

Encephalocentrist theories do not rely on general principles regarding the relations between wholes and their parts. Instead, many of them seem to be informed by an *inference to the best explanation*: As regards the specific case of *cognitive* activities, their being performed by a whole animal is best explained by there being a part of that animal which performs cognitive activities. In the eyes of encephalocentrists, empirical evidence demonstrates that statements like (2) or (2^*^) provide the best or perhaps even the only credible explanation of facts like (1)^6^.

It is not that encephalocentric predications lead to fallacies. *Au contraire*. An inference to what encephalocentrists regard as the best explanation of cognition leads to applications of psychological predicates that Neo-Aristotelians regard as illicit. Ironically, this conforms to a sense of “fallacy” that differs from the logical one they employ: “a mistaken or delusory belief or idea, an error, esp. one *founded on unsound reasoning*” (OED, my emphasis).

The real allegation against encephalocentric predications is that they evince a *category mistake*. Ascribing cognition to the brain is not just unwarranted or false, it is bereft of sense. It applies mental predicates or concepts to things that are *not even potential candidates* for satisfying these concepts. The cognitive capacities and performances invoked in (2) can only be meaningfully attributed to the animal as a whole, and not—save metaphorically—to its parts^7^.

We must keep this in mind when it comes to the justification of the Nonsense View. That view is not vindicated by the mereological principle. The latter is trivially true, since “apply only” is here intended as “are applicable intelligibly.” It leaves open the contested issue: *why* should psychological predicates be meaningfully ascribable only to whole animals, not to their brains?

The argument behind the Nonsense View is best reconstructed as follows:

**Criterial Premise**: The ascription of a psychological predicate “F” to an object x is meaningful (and hence truth-apt) only if x can satisfy the criteria for being F.

**Behavioral Premise**: The criteria for an object x satisfying a psychological predicate “F” are human behavior or behavior resembling it on the part of x.

**Wittgensteinian Conclusion:** The ascription of a psychological predicate “F” to an object x is meaningful only if x can engage in human(-like) behavior.

**Differentialist Premise**: Neither the brain nor its parts can engage in human-like behavior.

**Nonsense Conclusion:** The ascription of a psychological predicate “F” to the brain or its parts is not meaningful (and hence not truth-apt)^8^.

The argument is valid. However, all three premises require clarification and vindication, which will be provided in the next three sections.

## Psychological Concepts and Criteria

In ordinary parlance, criteria are *ways of telling* whether something satisfies a predicate “F,” and hence evidence for a claim of the form “x is F.” That invites the suspicion that the Criterial Premise is merely “epistemological.” It insists that there must be ways of finding out whether x satisfies F; but this has no bearing on the “ontological” issue of whether x is indeed F (Searle, 2007, 104–5). However, the requirement formulated in the Criterial Premise is *semantic*. In the Wittgensteinian employment of the term, the criteria for x being F are evidence of a particular type, “*logically* good evidence.” Unlike inductive evidence, criteria are connected to x being F *conceptually* rather than through factual correlations established by experience. “F” would not mean what it does unless their fulfillment by x counted in favor of “x is F.” “The criterial grounds of the ascription of a psychological predicate are partly constitutive of the meaning of that predicate” (Bennett and Hacker, 2003, 83).

Perhaps ontological questions are prior to epistemological ones. Yet semantic questions are prior to both, since matters of meaning antecede matters of knowledge *and* of fact. There can be true or false, justified or unjustified, answers to the question “Is x F?”—“Does the brain cognize?”—only if that question is meaningful to begin with. That presupposes that the meaning of “F” has been determined at least provisionally. By the same token, there can be inductive evidence for x being F only if that condition is met.

Nevertheless, the semantics behind the Criterial Premise appears unduly verificationist^9^. Why should the meaningfulness of psychological predicates require criterial *evidence* that is available to us even in principle? But the Criterial Premise does not assume that we need to know *how to acquire evidence*, even under optimal conditions. It merely presupposes that it should be possible to *specify what such evidence would consist in*. Still, why can't one make do with specifying *application conditions* in the style of truth-conditional semantics? Now, such specifications can take various forms. One is disquotational, and follows the pattern “x satisfies the predicate “F” iff x is F”. Applied to our case, this yields:

(5) The brain satisfies the predicate “cognizes” iff it cognizes.

Their popularity in formal semantics notwithstanding, however, statements like (5) do not properly *explain* the predicate quoted on the left-hand-side. They do not provide a *standard* for distinguishing correct and incorrect applications of “cognizes.” And knowing statements like (5) is neither necessary nor sufficient for *understanding* “cognizes.”

A second way of specifying application conditions is less vacuous.

(6) The brain satisfies the predicate “cognizes” iff it forms beliefs and desires on the basis of gathering and processing information

But in actual linguistic practice, even (6) would not count as an adequate explanation of “cognizes,” if none of the explanantia on the right-hand sides could be somehow *operationalized* somewhere along the line. By the same token, someone who could only offer such explanations while being clueless about what kind of evidence *might count* for or against the fulfillment of the *explanantia* would at most be credited with a partial understanding^10^.

Finally, *even if* the impossibility of specifying possible evidence for or against the satisfaction of a psychological predicate being satisfied were no bar to its being meaningful, it would deprive the predicate of any point in theories concerning the nature and causes of behavioral and mental phenomena that are even partly empirical. Encephalocentrism would be *hollow* as an approach in cognitive science.

Even in its philosophical manifestations, encephalocentrism aspires to making contributions to empirical theory formation. Unsurprisingly, therefore, it does not founder at the *general* semantic hurdle posed by the Criterial Premise. Encephalocentrism allows for evidence that the application conditions of psychological terms are fulfilled. Indeed, it positively specifies that this evidence includes data concerning neurophysiological goings-on. The real crux concerns the semantics of *mental expressions* in particular. What are the *pertinent criteria*, the criteria which give meaning to our psychological expressions?

## Criteria and Behavior

This question leads on to the Behavioral Premise. It rightly notes that in both everyday and scientific practice we ascribe psychological predicates to others on the basis of how they are disposed to behave. These are the criteria—the evidential grounds—for the fulfillment of these predicates. Nonetheless, in concluding that these evidential grounds are “partly constitutive” of the meaning of psychological predicates, Bennett and Hacker seem to “confuse the behavioral criteria for the *ascription* of psychological predicates with the *facts ascribed by these* psychological predicates” (Searle, 2007, 103). The application conditions for predicates like “x cognizes” and the truth conditions for statements like (1) concern the mind rather than behavior. By a similar token, to say “Mary is in pain” is not to say “Mary manifests pain behavior.”

However, Bennett and Hacker deny explicitly that “the psychological predication is equivalent in meaning to the behavioral description the truth of which warrants its ascription (sic)” (2003, 82n35). “Criteria for the application of such a predicate are distinct from its truth-conditions—an animal may be in pain and not show it or exhibit pain behavior without being in pain” (2007, 210-11n18). The behavioral criteria for mental phenomena are “defeasible,” subject to countervailing evidence (2003, 82–3).

Unfortunately, a puzzle remains. According to the Neo-Aristotelians, behavioral criteria are not just partly constitutive of the meaning of psychological predicates, they provide “constitutive grounds” for applying these predicates (Smit and Hacker, 2014, 1081). At the same time, they acknowledge that x can, for instance, be in pain without displaying pain behavior and that x can display pain behavior without being in pain. But in that case pain behavior does not *constitute* being in pain even partly, since pain behavior is not even a *necessary* condition for pain.

The resolution of the puzzle is to reconceive the conceptual relation between mind and behavior. First, behavioral criteria are not just defeasible but also diverse and context-sensitive. What counts as a manifestation of a mental state by one subject on one occasion, may not for another subject or another occasion. And what someone is disposed to do as a result of being in a particular mental state also depends on her other mental states (Geach, 1957, 8; Glock, 1996, 50–8, 93–7). Secondly, “constitutive grounds” are not facts that constitute the *phenomenon* of x being in a psychological state, but simply *non-inductive evidence* for x being in that state. At the same time, it is *constitutive of the meaning* of a psychological predicate that *there are* behavioral patterns licensing its application independently of induction. Our psychological terms *would not mean what they do* if they were not bound up with *some* behavioral criteria or other, however diverse, context-dependent and defeasible. The capacity approach explains why this is so. Mental concepts have an essential connection to potentialities (dispositions, abilities). “Pain” would not mean what it does unless certain forms of behavior counted as manifesting pain in particular circumstances^11^. And it is part of psychological terms in general that they have some such manifestation. We would have no use for these expressions if they didn't^12^.

This take on the Criterial and the Behavioral Premise suffices to support the Wittgensteinian Conclusion and to put paid to strong encephalocentrism. If, for example, we started to ascribe cognitive terms like “x perceives” or “x is intelligent” exclusively on neurophysiological grounds, in complete disregard of x's capacities to respond to its environment and to solve problems, these expressions would have changed their meaning. By the same token, although one can truthfully ascribe intelligence to a subject that does not manifest it, one can ascribe intelligence meaningfully—truly or falsely—only to a subject *capable* of behaving intelligently, a subject for which something counts as manifesting intelligence.

## Behavior and the Brain

Repudiating strong encephalocentrism is compatible with ascribing mental properties to the brain and its parts *as well*. For it leaves open whether the Differentialist Premise holds. Can the brain and/or its parts behave in a way that satisfies the criteria of cognition? Dennett answers in the affirmative. He subscribes to the Wittgensteinian Conclusion of *Investigations* §281. At the same time, he denies that this precludes attributing mental attributes to neurophysiological phenomena. For

[…] brains and their parts *do* ‘resemble a living human being (by behaving like a human being)'—and this resemblance is sufficient to warrant an adjusted use of psychological vocabulary to characterize that behavior (Dennett, 2007, 78).

Dennett admits that it would be illegitimate to attribute “*fully fledged*” mental phenomena to the brain parts (Dennett, 2007, 87). That would be to confuse the “personal” level of explanation which is “non-mechanical” with the “subpersonal” level which is “essentially mechanical” (Dennett, 2007, 78–9, 93). Nevertheless, one can attribute an “attenuated sort of belief and desire,” stripped of many of their everyday connotations. “Just as a young child can *sort of* believe that her daddy is a doctor (without full comprehension of what a daddy or a doctor is), so a robot—or some part of a person's brain—can *sort of* believe that there is an open door a few feet ahead, or that there is something amiss over there to the right, and so forth” (Dennett, 2007, 87–8).

Dennett maintains that adopting such an “intentional stance” toward neurophysiological entities is a highly fruitful research programme that allows cognitive neuroscience to explain the foundations of our cognitive capacities. “Far from it being a *mistake* to attribute hemi-semi-demi-proto-quasi-pseudo intentionality to the mereological parts of persons, it is precisely the enabling move that lets us see how on earth to get whole wonderful persons out of brute mechanical parts” (Dennett, 2007, 88–9). This response faces two rejoinders. First, there is the need of explaining what *sort of* cognizing amounts to, not to mention “hemi-semi-demi-proto-quasi-pseudo intentionality.” Dennett's allusion to the attenuated sense in which a small child can believe that her daddy is a doctor does not absolve him of that requirement. While the child cannot satisfy all of the criteria for holding such a belief, it can satisfy *some* of them (Bennett and Hacker, 2007, 141). Furthermore, she can fully satisfy criteria for believing *simpler things*, such as that there is a toy car in the room. Neither point holds of the brain or its parts.

Secondly, even if it made sense to credit sub-personal instances with cognition, wouldn't this only push further back the problem of explaining personal instances? One would then need to explain the representational capacities of these postulated homunculi, which engenders a vicious regress^13^. Now, the vacuity of explanations of human personal cognition by reference to sub-personal equivalents of human cognizers is acknowledged on all sides. That is why Dennett's “homuncular functionalism” invokes hierarchically structured “ever more stupid” intentional systems of a neurophysiological kind (Dennett, 1994, 240). Events at level E^n^ (cognition at the personal level) are explained by events at E^n−1^, the latter by reference to events at E^n−2^, etc. The aim is to discharge the explanatory task without embarking on an infinite regress, through a finite number of steps terminating in a level of completely non-intentional mechanisms.

Bennett and Hacker (2003, 141–7) recognize that there are levels of explanation between psychological descriptions of the whole animal and neuro-chemical descriptions of (parts of) the brain. They brook information-theoretic descriptions of (clusters of) neurons. They do not consider all the different notions of information currently on the market (see Adriaans, 2018). Nevertheless, they are right to distinguish “engineering information” from “semantic information.” While the latter consists of true propositions that can be apprehended—believed, known—by an epistemic subject, the former concerns non-epistemic phenomena such as the probability of a datum and the freedom of choice in transmitting a signal.

There is a contrast between information as data or knowledge gained, possessed, and employed by whole animals on the one hand, mathematical constructs used to explain the causes and effects of neuro-chemical signals on the other. For this reason, the homuncular strategy does not address the crux of the debate: Is the application of psychological predicates (e.g., “possesses semantic information”) to *anything other than whole subjects* starting with levels E^n−1^ conceptually licit in the first place?

*If* it is not, if E^n−1^ is amenable only to predicates like “processes engineering information”, the attempt to causally explain phenomena at E^n^ through applying such predicates at E^n−1^ lacks sense and *a fortiori* explanatory power. The same holds for explaining (1) through (2) and (2^*^). By implication, explaining what (2) and (2^*^) *mean* by saying that they record the best causal *explanans* for (1) also fails. That Mary's brain cognizes—as of (2)—cannot mean that its cognizing causally leads to Mary cognizing, because there is no such thing as her brain cognizing. A related problem afflicts (2^*^). Either Mary's brain “sort of cognizing” is supposed to be a *genuinely cognitive and epistemic* episode; in that case we are facing the intelligibility question all over again. Or it is supposed to be an episode beneath that threshold, notably a neurophysiological or information-theoretic process; in that case, a causal explanation is on offer, yet it does not involve a contested encephalocentric predication.

A hierarchy of increasingly “dumb” homunculi raises questions about conceptual differences for each transition between levels of explanation. In consequence, the encephalocentrist faces a dilemma. *Either* he discharges the obligation to explain what *sort of* cognizing by parts of the brain amounts to through further mentalist and epistemic vocabulary. In that case we are back at square one, since it remains unclear what the application of such vocabulary to parts of the brain amounts to. *Or* he explains it by saying that it means that processes in the brain of a neurophysiological or information-theoretic kind causally explain the cognizing of the whole person. In that case the message is clear enough.

(2^*^)Mary's brain/a part of Mary's brain *sort of* cognizes

would amount to

(2#)Mary's brain/a part of Mary's brain undergoes a neurophysiological or information-theoretic process & that process is causally responsible for (enables) Mary's cognizing.

On that construal, (2^*^) involves a dual metonymical transfer, from a whole to its part, and from an effect to its cause. But then the attribution of mental properties to sub-personal instances is merely a figure of speech; indeed, it is a dispensable shorthand. The only remnant of encephalocentrism is the contention that the brain is causally responsible for cognition (see section Is the Brain the Organ of Cognition?).

## Technical Uses and Metaphor

There are alternative ways in which cognition might be attributed to parts of the brain in an attenuated, non-literal way. The first is that the use is technical. In that case, we would be dealing with *polysemes* of psychological expressions. But it would not just be incumbent on neuroscientists to explain their technical uses; they would also have to keep these uses apart from non-technical ones. Now, our mental concepts as applied to whole flesh-and-blood subjects determine the primary topics of philosophy of mind and cognitive science. The fundamental questions concerning mind and cognition are phrased in *extant, non-modified* vocabulary; indeed, mental idiom is first and foremost part of everyday discourse. We want to know, e.g., whether animals or brains think or are conscious in our sense of these terms, not in a sense introduced by new-fangled theories. To be sure, cognitive science also discovers and conceptualizes novel phenomena. And in tackling the initial topics it likewise introduces new concepts. For instance, the explanation of perception must employ technical concepts from a variety of areas, ranging from behavioral psychology to biochemistry. Yet statements couched in everyday terms like “Maria *saw* that Frank had put on weight,” “Sarah *listens* to the *Eroica*,” “One can *smell* the wild strawberries,” “The sense of *taste* is not affected by old age,” “In the Müller-Lyer illusion two lines of equal length *appear* to be of unequal length,” etc., pick out the basic *phenomena that the science of perception seeks to explain*.

Small wonder, then, that in presenting, interpreting, and drawing conclusions from their empirical data concerning perception, cognitive scientists do not uniformly stick to technical terminology. Instead, they often employ everyday terms like “representation,” “symbol,” “map,” “image,” “information” or “language” in ways which *either* remain unexplained *or* illicitly combine their ordinary uses with technical ones with an entirely different semantic import.

Any verdict to this effect needs to be sustained through painstaking analysis of individual cases. This cannot be undertaken here^14^. A general moral can be drawn nonetheless. The *explicit introduction* and *consistent employment* of homonyms of established psychological terms may be liable to cause confusion, yet it is unexceptional in principle. By that very token, however, it not merely avoids category mistakes; like the metonymical transfer in (2#), it eschews any encephalocentrism that the Nonsense View or situated cognition would have to resist. If belief^*^, knowledge^*^, and information^*^ are used on the basis of neurophysiological or information-theoretic criteria, they apply to the *explanantia*—the phenomena which explain, respectively, the formation of beliefs, the acquisition of knowledge and the possession of information. Yet they do *not* univocally apply to these un-asterisked *explananda*.

A second way of attenuating the sense of an expression is metaphor. Weak encephalocentrists try to assuage doubts by pleading that applications of cognitive terms to the brain and its parts are metaphorical (Blakemore, 1990; Searle, 2007, 112; Dennett, 2013). Metaphors serve a substantial heuristic function. They draw attention to features of the phenomenon to which they are applied by highlighting similarities with other phenomena. That is why they were traditionally regarded as abbreviated comparisons, (rightly so, see Schroeder, 2004). Metaphors are invaluable for many purposes. Still, if they are to lead our thinking in fruitful directions, they must be recognized as such.

This has important implications for allegations that encephalocentrists commit category mistakes. On the one hand, they must be made out separately for each contested case. On the other hand, it does not suffice for the accused to plead that they mean certain expressions to be taken metaphorically. They face two further demands. First, they must specify the respect in which the neurophysiological subjects of cognitive predicates resemble the personal ones. Secondly, that resemblance must suffice for the purposes—explanatory or justificatory—which the allegedly metaphorical use is meant to fulfill.

A metaphor draws attention to certain aspects of a phenomenon. But it contributes to an explanation only to the extent to which that phenomenon shares relevant features with things to which the metaphorical term applies literally. The purpose of using metaphors in an explanatory capacity must be to *compare* the explanandum with phenomena belonging to the literal extension of the term. The potential explanation is that there is an analogy between the explanandum and these phenomena.

## Analogies

At this point metaphors trade on a third way of attenuating the sense of an expression, namely to extend it by way of *analogy*. Paradigmatic examples include the extension of hydrodynamic notions such as “current” to the theory of electricity. Attributions of cognition to the brain are often explicitly defended as appeals to analogy. The idea fuels Gregory's animadversions to “semantic inertia” (Gregory, 1987, 242–3) and Dennett's insistence that sub-personal processes in the brain are “*strikingly like*” personal cognitive processes (Dennett, 2007, 86). In response, Bennett and Hacker complain that the “application of psychological expressions to the brain is not part of a complex theory replete with functional, mathematical relationships expressible by means of quantifiable laws as are to be found in the theory of electricity” (Bennett and Hacker, 2003, 77). However, Figdor (2018, ch. 3) argues that recent analogical theories revolve around precise models of relationships at a sub-personal level, nonetheless characterized in cognitive terms. The “temporal difference model” of reinforcement learning and the “predictive coding hypothesis” explain cognitive capacities and processes by exploiting “quantitative analogies” between neurophysiological phenomena and personal cognition, employing mathematical models and equations (2018, 31).

Nevertheless, there is a contrast between these novel theories in cognitive science and explanations of mechanical terms in physics, like “force” or “work.” The latter diverge, often radically, from the everyday understanding of these terms. Yet they do so across the board, and in a clear-cut and mathematically precise manner, one giving rise to quantifiable laws^15^. What is more, they are patently fruitful. Figdor maintains that the aforementioned quantitative models are “highly confirmed.” But there is no consensus concerning their precise interpretation, predictive accuracy or fertility. Indeed, many cognitive and life scientists concur with Bennett and Hacker's condemnation of the mereological fallacy^16^.

There are also problems of principle with the analogy defense, even as applied to such quantitative theories. First, when cognitive labels like “learning” and “prediction” are not just quantitatively regimented but also transferred to neurophysiological subjects, they change their meaning, just as, e.g., “current” changed its meaning when transferred from liquids to electrodynamic phenomena. Secondly, in what does the analogy between the categories of these theories and the personal mental ones consist in? To avoid a *petitio* in favor of encephalocentrism, the sub-personal processes would have to be described in uncontentious terms, which means in terms that are either neurophysiological or information-theoretic. In that case, however, the analogies are of a *purely formal* or *structural* kind: certain mathematical models apply equally to both. In other respects, the employment, in particular the combinatorial possibilities and the inferential patterns, of the sub-personal expressions is far remote from that of the personal ones. It makes no sense to speak of columns of brain cells as inferring, calculating, predicting or perceiving *while going for a walk* (or for the purpose of foraging and preparing food)—these are not activities neurons can engage in.

More importantly, the incongruity also holds for psychological contexts. Neither the brain, nor one of its parts, nor neural tissues nor individual neurons can act on beliefs in conjunction with desires or goals; they do not show surprise when a belief or prediction turns out to be false, they do not modify their beliefs on account of the deliverances of their senses; they cannot be distracted in their cogitations by perceptual inputs, nor can cogitative assumptions taint their perceptions (“cognitive penetration”); they are not overwhelmed by emotions when experiencing joyful or sad situations; they do not avow their beliefs; they do not communicate their predictions and consider them in the light of objections by others. In short, what the mathematical models are capable of capturing in a way that is semantically and methodologically controlled and potentially fruitful once more concerns the causal enabling conditions of cognition, not features they share with cognition at the personal level.

But what of encephalocentrists who are prepared to go the whole hog? They might insist that brains, their parts, strata of neurons, individual neurons, etc., engage in all these mental activities. Not, of course, in the open-air but—taking work from home to extremes—inside the skull and in a neuro-chemical medium. Thus, Figdor deliberately casts to the wind a distinction by predictive processing theorists. To signify a mismatch between a predicted signal in the brain and the actual input, they use the technical neologism “surprisal” rather than the everyday “surprise.” They should use the latter in a literal sense, she avers; for in both the personal and the sub-personal case there is a “discrepancy between an expectation and an observed actuality” (Figdor, 2018, 56). However, this obviously begs the question by assuming that there is a clear-cut similarity between “expectation” and “observation” at the personal and the sub-personal level. One cannot explain the use of one terminology—psychological idiom–in an area in which it is obscure and contested (the brain) through the use of another terminology—such as the idiom of behavior—in the same area, if that application is equally obscure and contested.

Admittedly, neurophysiological entities can behave, in the sense of causing change. But this holds of inanimate substances as well. It does so precisely because not all activity is psychological or guided by cognition. Perhaps one can mathematically model neural activity accurately in ways formally analogous to cognitive processes like predicting and adjusting expectations. Yet this no more shows that neurons actually engage in such cognitive activities than the fact that one can model the movement of planets through Kepler's laws shows that the planets deliberately follow these laws.

This last comparison highlights a final challenge facing hard-boiled encephalocentrists. Structural analogies with either human cognition and activity in this comprehensive sense are not confined to animate systems; they can be detected across the physical world. Radical encephalocentrists must be prepared to ascribe cognition, plus all of the concepts connected to it, to things on grounds of similarities, however thin and abstract, with personal subjects of cognition. If that were legitimate, what could bar ascribing them to any physical phenomenon whatever? Radical encephalocentrism threatens to lapse into panpsychism^17^.

## Philosophers, Nobel Laureates, and Nonsense

According to Figdor, applications of all psychological concepts, however sophisticated, to animate subjects of any kind, however primitive, are not just legitimate, they are to be taken at face value. This semantic doctrine—“Literalism”—goes along with “Anti-Exceptionalism,” according to which “the relevant scientific evidence shows that psychological capacities are possessed by a far wider range of kinds of entities than often assumed. Literalism claims that, in contexts standardly interpreted as fact-stating, uses of psychological predicates to ascribe capacities in this wider range are best interpreted as literal with sameness of reference. Anti-Exceptionalism is the metaphysical position that underwrites the claim of sameness of reference” (Figdor, 2018, 5–6).

From this perspective Figdor attacks the Nonsense View. She quotes Bennett and Hacker: “If a form of words makes no sense, then it won't express a truth.” She then turns their modus ponens into a modus tollens: ascriptions of psychological predicates to the brain “are expressions of truths (or empirically statements), so the Nonsense view fails” (Figdor, 2018, 98). But this tactic presupposes that the contested predications make sense. Figdor intimates two interconnected arguments for this presupposition. One is that these predications differ from clear-cut cases of nonsense like semantic anomalies. The other is that philosophers are not entitled to condemn pronouncements by Nobel laureates as nonsensical.

Both arguments ignore a central aspect of the Nonsense View. It is based on the idea that there are different kinds of nonsense or conceptual incoherence. Not all of them are gibberish or semantic anomalies. Certain types of philosophically relevant nonsense result from failure to pay heed to subtle features of concepts in the course of complex lines of reasoning, often as a result of being misled by powerful pictures and intellectual pressures. We are dealing with “latent” rather than “patent” nonsense (Wittgenstein, 1953, §464). This holds especially of category mistakes. There is no reason why scientists, however accomplished, should be immune to such confusions, especially when it comes to spelling out the implications of neurophysiological data for the psychological phenomena to be explained. Conversely, there is some reason to believe that philosophers can acquire both the conceptual sensitivity and the dialectical acuity to rectify such confusions. Even if that hope were overly optimistic, the numerous contradictions, paradoxes and antinomies that have been derived from apparently innocent premises and solid empirical findings provide ample evidence that conceptual inconsistencies and category mistakes need not lie open to view. The Nonsense View has it that encephalocentrists fall prey to such far from trivial mistakes. This results in a type of nonsense, since it cannot be spelled out coherently what encephalocentric predications mean in the context of the encephalocentrists' own explanations and arguments. Even if linguistic nonsense were the wrong category for category mistakes, the charge that encephalocentrism commits such mistakes would remain damning; and it cannot be dismissed simply by noting that famous scientists are not in the habit of talking gibberish.

Figdor acknowledges that Bennett and Hacker are right in complaining that cognitive neuroscientists often cause “confusion” by playing “fast and loose” with psychological predicates, notably by “defining terms in orthodox behaviorist manner and then drawing inferences that presuppose a cognitive interpretation” (Figdor, 2018, 104, 96, 98). But for her such lapses are confined to “the public communication of neuroscience.” Bennett and Hacker, she contends, take account of “works intended for popular audiences,” “they do not engage with the relevant scientific literature.” This allegation misfires in two respects. First, it is incompatible with another dig Figdor takes at Bennett and Hacker, namely that “their view entails that Nobel prize-winning neuroscientist are writing nonsense in papers that helped garner them the prize” (Figdor, 2018, 94). Leaving aside the whiff of an appeal to Nobel authority, *if* the points raised by Bennett and Hacker indeed concerned only popular writings by neuroscientists, they could not entail any conclusions about their scientific publications, least of all if the two genres were as remote from each other as Figdor has it. Secondly, Bennett and Hacker cite numerous articles and books aimed at specialists (e.g., Bennett and Hacker, 2003, 75–81; Bennett and Hacker, 2007, 154–6). This leaves the worry that their targets are “often …. somewhat dated in neuroscience terms” (Figdor, 2018, 91). But on the same page, she concedes that “some recent peer-reviewed work in cognitive neuroscience … involves similar usage (or misusage).” So Figdor's allegation that Bennett and Hacker miss the “forest [serious neuroscience] for some epistemically inconsequential bushes nearby [popular neuroscience]” (Figdor, 2018, 100) is itself off the mark.

## Elucidation vs. Revision

Figdor is nevertheless right in noting that “Bennett and Hacker and I are writing at cross purposes” (Figdor, 2018, 94). The reason is not, however, that she engages with respectable science whereas they target an Aunt Sally by restricting themselves to popularizations. It is rather that they are concerned with our *extant* concepts, whereas she explicitly charts and promotes a process of “conceptual revision” (1). On occasion, she acknowledges the radical nature of the proposed conceptual change (e.g., Figdor, 2018, 29). But she also maintains: “the rules for psychological predicates *have* changed” (Figdor, 2018, 96). This may hold to some extent for their application to non-human animals and computers. But the explicit conviction that these predicates apply non-metaphorically to organs, plants, and micro-organisms has not become entrenched in either quotidian or scientific discourse. In any event, the Nonsense View explicitly addresses our psychological concepts before the revolution propagated by Figdor. More importantly still, the crux of the matter is whether the extension (whether *fait accompli* or envisaged) is indeed governed by rules that are both consistent and do not simply change the topic. In deploring conceptual back-sliding in popular neuroscience Figdor acknowledges willy-nilly both that this demand is legitimate and that it is frequently violated.

Like Figdor's Literalism in general, her rejection of the Nonsense View is based on Anti-exceptionalism. It depends on a realist semantics according to which scientific discoveries inform us not just about the actual extensions of psychological expressions, but also about their intensions or meanings. The rules have changed, this semantics implies, in the direction of capturing the real essences of psychological phenomena. In the wake of Kripke and Putnam, this has been a dominant view, and scrutinizing it is beyond the current remit. However, realist semantics has been explicitly criticized by proponents of a Nonsense View, especially as regards psychological expressions (Hacker, 1996; Glock, 2017). It is at odds with the “Wittgenstein-inspired rule-based semantics” that underlies their argument. This undermines Figdor's verdict that the Nonsense view fails even on its own terms (Figdor, 2018, 96, 100).

Empirical discoveries can show that the extension of extant concepts is different from what we used to think. Scientific theory revision, especially of a revolutionary kind, can also motivate conceptual change, the modification or replacement of those concepts. But, in line with the Criterial Premise, these concepts are determined by the criteria by which we decide *whether* something belongs to the extension. Therefore, scientific discoveries cannot show that our “traditional anthropocentric *grounds* for establishing the proper extensions of psychological predicates” are incorrect (Figdor, 2018, 104; my emphasis). As Davidson reminded us, “Our concepts are ours” (Davidson, 1999, 19). They play a role in our cognition, serve our epistemic needs and interests, and are geared to our capacities. To that extent, our extant *mental concepts* are anthropocentric; yet they are none the worse for that! Moreover, it does *not* follow that it is anthropocentric to insist that these concepts preclude application to brains and their parts.

Finally, the Wittgensteinian semantics undergirding the Nonsense view is more congenial to situated cognition than realist semantics. Situated cognition treats psychological concepts as means of making sense of others and ourselves, rather than as metaphysical lasers that “carve nature at its joints,” in Plato's striking phrase.

## Is the Brain the Organ of Cognition?

In another area, Neo-Aristotelianism is congenial to situated cognition up to a point. It shows that most of our extant psychological terms apply literally to whole subjects rather than their parts. This removes the pressure to *locate* cognition within a subject's skull. Questions like “Where did A perceive X/recognize that p/decide to Φ?” are answered by locating A in her environment (Bennett and Hacker, 2003, 128; Bennett and Hacker, 2007, 142–3). But Neo-Aristotelians take the rejection of encephalocentrism one step further. They deny that the brain is the *organ* of cognition.

The stomach can be said to be digesting food, but the brain cannot be said to be thinking. The stomach is the digestive organ, but the brain is no more an organ of thought than it is an organ of locomotion [Fn25: One needs a normally functioning brain to think or to walk, but one does not walk *with* one's brain. Nor does one think *with* it, any more than one sees or hears with it]. If one opens the stomach, one can see the digestion of the food going on there. But if one wants to see thinking going on, one should look at the (sic) *Le Penseur* …, not at his brain. All his brain can show is what goes on there *while he is thinking* (Bennett and Hacker, 2007, 143).

The brain is not an organ with which we can do anything, though we cannot do anything without a brain (Smit and Hacker, 2014, 1082).

The first, and uncontested, point to note: the brain is an organ, though complex in anatomical and physiological terms. In biology, an organ is a group of tissues that performs certain functions. Like many organs (skin, liver, sexual organs), the brain fulfills a variety of functions.

Secondly, Bennett and Hacker deny that enabling cognition is one of these functions (Bennett and Hacker, 2003, 152). But their argument at most shows that the brain's function is not to enable cognition *on the part of the brain*. Furthermore, the denial is at odds with their observation that “the brain … enables the animal to see a visible scene” (2003, 139). Finally, it ignores the biological fact that enabling cognition is a crucial contribution that the central nervous system makes both to the well-being of individual animals and the adaptive advantage that its emergence conferred in evolutionary history.

Thirdly, Bennett and Hacker (2007, 135) acknowledge that one cannot cognize without the brain. To them, this does not show that the brain is the organ of cognition. For one cannot run without the brain either, and no one would say that the brain is the organ of locomotion. There is a difference, however. Neurophysiological processes and the proper functioning of the brain are the *proximate* causal enabling conditions of cognition. By contrast, the brain's causal relation to the movement of our locomotive organs is distal, mediated by motoric nerves, sinews, and muscles. Therefore, acknowledging that the brain is the organ of cognition does not commit one to maintaining that it is the organ of locomotion. In the terminology of the capacity approach, it is to say that the brain is the *vehicle* of cognition. It is that physical component of an animal which is directly responsible for its possessing cognitive capacities and causally involved in the exercise of those capacities.

Fourthly, Bennett and Hacker bluntly deny that we do anything *with* our brains. To be sure, we do not have direct voluntary control over what happens in our brains the way in which—through neurophysiological mechanisms like proprioception and motor nerves—we have control over the movement of our limbs. But as they recognize, this holds of other organs like the stomach as well. And we *do* digest with our stomachs.

Fifthly, established parlance suggests something analogous for cognition and the brain. According to Smit and Hacker “Use your brain!” simply means “Think!.” “It no more signifies that we think with our brains than “I love you with all my heart” signifies that we love with our heart” (Smit and Hacker, 2014, 1089). But we employ “Use your brain!” to signify “Think!” *because we assume* that it is your brain that must operate properly for you to think. By contrast, we do *not* assume that your heart plays a special proximate role in enabling your emotions. “My brain isn't working properly today” is *not* a metaphor. It is on a par with “My stomach isn't working properly today.” Both allude to causal factors influencing the enabling conditions of, respectively, my intellectual and metabolic capacities. That is why there is nothing *conceptually* amiss with trying to improve one's intellectual performance through “cognitive doping,” imbibing drugs with neurophysiological effects.

Sixthly, that the brain is the organ of cognition is a major objection to 4E cognition (see Adams and Aizawa, 2008). Acknowledging this point and granting that there is a sense in which *we* think *with* our brains does not amount to backsliding into encephalocentrism. It no more entails that it is the brain that cognizes or that cognition occurs in the brain than the fact that our legs are the organs or vehicles of running entails that it is the legs that run on their own or that running occurs in our legs–as any marathon runner will testify.

Seventhly, Neo-Aristotelians are dead right that we cannot observe thinking in the brain. For one thing, the connection of cognition to neurophysiological phenomena is contingent, by contrast to its connection to behavioral capacities. For another, since cognizing is something done by whole subjects, it can only be observed by noting what these subjects do and are capable of doing. Nevertheless, in the brain we can observe neuro-chemical processes (indirectly, e.g., through fMRI scanners detecting rates of metabolism), and these processes do not merely accompany cognition, as Neo-Aristotelians have it, they *causally enable* it.

Eighthly, we can indeed observe digestion taking place in the stomach. Still, the contrast to cognition and the brain depends partly on how one conceives of digestion. At one level, digestion consists of chemical processes that take place in the gastrointestinal tract. But even a purely physiological account will have to include its interaction with other organs (liver, kidneys). In so far as digestion is the metabolic process that supplies energy to the whole organism, it is something that the whole organism engages in.

## Conclusion

Situated cognition adopts such a wider, loosely speaking ecological, perspective. The capacity approach can provide a conceptual framework for this paradigm. Our psychological vocabulary captures *neither* neurophysiological or computational *nor* genetic-*cum*-genomic *nor* evolutionary differences, all of which are accessible at best on the basis of sophisticated instruments and theories. Instead, it captures differences in the kinds of *behavioral* and *perceptual* capacities human beings are both interested in and have unproblematic access to. This is unsurprising, especially from the perspective of situated cognition. We are social and cooperative primates by nature. Our languages include mental terms because of our fundamental need to describe, explain, predict and otherwise understand the behavior and behavioral dispositions of other human and non-human animals, and because of the equally fundamental need to express ourselves to other humans. No room here for the inner glow sought by Cartesians, or the neural mechanisms that captivate encephalocentrists.

Instead of emphasizing the brain at the expense of whole animals and their capacities, both situated cognition and the capacity approach adopt a perspective that is more realistic, and more naturalistic to boot. Cognitive and biological phenomena reveal themselves only when we go beyond the brain and consider not just the whole organism, but also the way the organism exercises its capacities in the context of its physical and social environment, in accordance with its “form of life,” as Wittgenstein would put it.

Conversely, the capacity approach can profit from ideas and aspirations of situated cognition. Its chief merit lies in avoiding the misguided Cartesian riddle about how two ontologically distinct substances like mind and body can causally interact, since it recognizes that the former is not a substance to begin with. Contrary to some advocates (Maslin, 2006, 209–19), however, it does not thereby dispatch the mind–body problem *tout court*. For one thing, capacities require a causal substratum, implementation or vehicle. This poses a scientific challenge—facing cognitive neuroscience and information theory—of explaining precisely how the vehicle of mental powers—the brain—causally sustains the power.

For another, capacities are defined by reference to how they are exercised. These exercises in turn are events and processes that stand in relations of efficient causation. Therefore, the question remains of what role causation plays for the episodic behavioral, mental and neurophysiological phenomena through which mental capacities are actualized or implemented. It won't do to claim, for instance, that feeling a pain is simply the actualization of the mental capacity for sentience. That answer is unexplanatory, not just in a factual-cum-scientific but also in a conceptual-cum-philosophical capacity.

At the scientific level, one needs to confront the question: what type of causal relation obtains between certain mental events and cognitive capacities on the one hand, neurophysiological processes, and structures on the other? More specifically, how do various mechanisms have to combine causally to constitute a suitable vehicle of cognition? What kinds of information processing need to occur in the central nervous system to provide its possessor with what kind of perception or intelligence? What neuro-chemical mechanisms can sustain such a flow of information? On such issues conceptual analysis should interact with empirical theory-formation of the kind undertaken within situated cognition. This article aimed to vindicate the shared opposition to encephalocentrism on which such interaction could be based.

## Author Contributions

The author confirms being the sole contributor of this work and has approved it for publication.

## Conflict of Interest

The author declares that the research was conducted in the absence of any commercial or financial relationships that could be construed as a potential conflict of interest.

## Footnotes

^1^Adams and Aizawa (2008) use “contingent intracranialism” and Hohwy (2016) “neurocentrism” as labels for positions they defend.

^2^The exceptions concern Wittgenstein (Hutto and Myin, 2013; Kiverstein and Rietveld, 2015). There is as yet no comparison of situated cognition and Neo-Aristotelianism, and no discussion of their shared opposition to encephalocentrism.

^3^Thus Hohwy (2016) argues that (i) predictive processing, the view that “the brain minimizes its prediction error and thereby infers the states of the world” is “rapidly gaining momentum”; (ii) predictive processing implies a “neurocentrically skull-bound” picture of the mind; (iii) such a picture is incompatible with extended and embodied cognition. (i) is an understatement [one of many leading empirical neuroscientists relying on this paradigm is Dehaene (2020, ch. 2)]. (iii) is undeniable. And it is difficult to avoid (ii), unless one interprets talk of prediction and inferences as metaphors for information processing that causally enables cognitive processes without itself being cognitive. Consequently, for situated cognition there is a premium on showing that literal interpretations yield indefensible claims.

^4^Such accommodations include “extended functionalism” (Wheeler, 2010), theories incorporating environmsental factors into predictive processing (Clark, 2016), and Searle's view that we are “embodied brains” (2007, 119–21).

^5^For a discussion whether debates about the locus of cognition make a difference to *experimental* cognitive science see Kiverstein (2018, 23–4).

^6^This holds for pioneers of cognitive neuroscience: “We seem driven to say that such neurons have knowledge” (Blakemore, 1977, 91); “If we are capable of knowing what is where in the world, our brains must somehow be capable of *representing* this information” (Marr, 1980, 3); “It is an inescapable conclusion that there must be a symbolic description in the brain of the outside world” (Frisby, 1980, 8). It is also a guiding theme in contemporary research. “[T]he theory that the brain is a sophisticated hypothesis-testing mechanism … is meant to explain perception and action and everything in between” (Hohwy, 2013, 2). Regarding an alternative theory according to which the brain uses “semantic pointers” to combine sensory, motor, and verbal presentations, Thagard claims that it is “the result of an inference to the best explanation of the full range of relevant evidence” (Thagard, 2019, 15).

^7^For the sake of argument, I assume that category mistakes are nonsensical rather than conceptually false. See Glock (2015).

^8^See Bennett and Hacker (2003, 71, 83). Their version of the Criterial premise runs: “The criterial grounds of the ascription of a psychological predicate are partly constitutive of the meaning of that predicate.” My formulation eschews complications arising from their idiom of “criterial grounds” and “partly constitutive” (see section Criteria and Behavior) and from analytic functionalism, a position which accepts their premises while rejecting their conclusion.

^9^It is a “Wittgenstein-inspired rule-based semantics” (see section Elucidation vs. Revision). But it is not a “criterial semantics,” since these rules need not specify criteria in the sense explained in section Criteria and Behavior. Still, they should specify conditions of application in an informative way capable of guiding linguistic practice.

^10^Consider a similar case: someone who can specify necessary and sufficient conditions for satisfying “tadpole”—being an amphibian at the larval stage of its life cycle—without being able to indicate what conceivable evidence (dis-)confirms something being an amphibian or larva is not a fully competent user.

^11^Our use of psychological expressions is not guided by exemplars. The grounds for ascribing pain to x is not that x *resembles*, e.g., the Man of Sorrows. It is that x *behaves* in a way that, in x's current circumstances, is a paradigmatic *manifestation* of pain. They resemble the features that characterize proto-/stereotypes in providing evidence that is defeasible. Flying will not count as evidence for x being a bird if x suckles its young or is invertebrate. But the defeating conditions for behavioral criteria are more context-dependent. Whether sobbing counts as evidence for x grieving for someone can depend not just on x's current setting and behavioral pattern but also on x's past history.

^12^We nevertheless understand ascriptions of mental states and processes to someone who is completely paralyzed. For the exercise of mental abilities can have behavioral manifestations only if certain enabling conditions obtain. I am grateful to a reviewer for this point, which indicates how the capacity approach can strengthen Wittgenstein's reflections.

^13^Unlike justification, explanation is *not* conditional. One can explain E_n_ by reference to E_n−1_, *without* being able to adduce, even in principle, an explanans E_n−2_ (e.g. if E_n−1_ is the Big Bang). But that presupposes that events of *type* E^n−1^ are intelligible and uncontentious. According to the Nonsense View, purported explanations like (2) fail both conditions.

^14^That such oscillations occur is granted even by Figdor, see section Philosophers, Nobel Laureates, and Nonsense. A reviewer helpfully suggested that in psycho-linguistics and in priming experiments “X is inferred from Y” is often understood as “X is activated by Y.” This causal gloss causes confusion when combined with the familiar logico-epistemic one. For instance, when subjects are primed to draw inferences in that sense, areas in the right hemisphere are activated; from this it is concluded that the right hand hemisphere contributes to drawing inferences, without noting the switch to a causal sense (e.g., Mirous and Beeman, 2012).

^15^Figdor insists that laws are unnecessary for “respectable biological theories” (2018, 95). But the extent to which biology aspires to such laws is a bone of controversy (Ayala and Arp, 2010, Part I). Furthermore, laws had better be part of the “model-based extensions” of psychological concepts based on *quantitative analogies* which she invokes against the Nonsense View.

^16^Figdor (2018, 98–100) plays down this fact by maintaining that their agenda differs from that of the Nonsense View. Yet even if this were true, it would not show that their agreement is based on misunderstanding or that they accept encephalocentrism after all.

^17^Figdor denies that she is committed to panpsychism (Figdor, 2018, 9–10). Alas, she does not even intimate how it is to be avoided. Unsurprisingly, given that the similarities that connect human cognition to, e.g., information processing by bacteria, are so cheap that it is exceedingly difficult to draw a line.
